# Loganin Attenuates Rotenone-Induced Parkinsonism-like Features in Rats Through Multi-Target Neuroprotective Mechanisms

**DOI:** 10.3390/biomedicines14061195

**Published:** 2026-05-25

**Authors:** Peng-Yuan Chang, Mao-Hsien Wang, Yu-Ling Yeh, Kuo-Chi Chang, Hung-Sheng Soung

**Affiliations:** 1 Department of Neurosurgery, Tao-Yuan General Hospital, Ministry of Health and Welfare, Taoyuan City 330, Taiwan; 2 Department of Anesthesia, En Chu Kon Hospital, Sanshia District, New Taipei City 23702, Taiwan; 3 Department of Anesthesiology, Shin Kong Wu Ho-Su Memorial Hospital, Taipei 11101, Taiwan; 4 National Applied Research Laboratories, Institute of Taiwan Instrument Research, Hsinchu 300092, Taiwan; 5 Department of Chemical Engineering and Biotechnology, National Taipei University of Technology, Taipei 10608, Taiwan; 6 Department of Psychiatry, Yuan-Shan Branch of Taipei Veteran General Hospital, No. 386, Rongguang Rd., Neicheng, Yuanshan Township, Yilan 26604, Taiwan; 7 Department of Biomedical Engineering, National Defense Medical Center, Taipei 11490, Taiwan

**Keywords:** loganin, rotenone, selegiline, trigonelline, oxidative stress, mitochondrial dysfunction, catecholamines, Parkinson’s disease

## Abstract

**Background/Objectives:** Rotenone (RT)-induced neurotoxicity is widely used to model Parkinsonism-like nigrostriatal injury and recapitulates several PD-relevant pathological features, including oxidative stress, mitochondrial dysfunction, neuroinflammation, and dopaminergic neurochemical disturbance. Loganin (LG), an iridoid glycoside isolated from *Cornus officinalis*, has been reported to possess antioxidant, anti-inflammatory, anti-apoptotic, and neuroprotective properties. However, its protective effects in a unilateral stereotaxic RT lesion model have not been fully elucidated. This study aimed to investigate the neuroprotective potential of LG against RT-induced Parkinsonism-like pathology in rats and to explore the possible involvement of antioxidant-related signaling mechanisms. **Methods:** Adult male Wistar rats were randomly assigned to twelve experimental groups (*n* = 8/group), including control, sham, RT, sham + LG, RT + LG, RT + trigonelline (TG) + LG, and RT + selegiline (SL). RT was stereotaxically injected once into the right substantia nigra pars compacta (SNpc) on Day 0 to induce unilateral nigrostriatal injury. LG was administered orally once daily from Day 1 to Day 21 at doses of 3, 10, and 30 mg/kg. TG was given intraperitoneally 30 min before LG treatment, while SL served as a reference antiparkinsonian drug. Behavioral assessments and biochemical analyses were conducted to evaluate motor dysfunction, oxidative and nitrosative stress, endogenous antioxidant status, mitochondrial dysfunction, inflammatory and apoptotic responses in the SNpc, and striatal catecholamine disturbances. **Results:** RT lesioning produced significant motor deficits, oxidative and nitrosative stress, depletion of endogenous antioxidant defenses, mitochondrial dysfunction, inflammatory and apoptotic activation in the SNpc, and abnormalities in striatal catecholamine levels. LG treatment significantly attenuated these pathological changes, with more pronounced protective effects observed at 10 and 30 mg/kg. Co-administration of TG partially weakened the beneficial effects of LG, suggesting the possible involvement of antioxidant defense-related signaling while not providing direct proof of a single pathway. SL also ameliorated RT-induced behavioral and biochemical abnormalities. **Conclusions:** These findings suggest that LG confers multi-target neuroprotective effects against RT-induced Parkinsonism-like features in rats. The protective actions of LG were associated with attenuation of oxidative stress, mitochondrial dysfunction, neuroinflammation, apoptosis, and catecholaminergic disturbances. Because the pathway analysis remains pharmacological and indirect, additional studies using direct molecular validation are warranted before LG can be considered a disease-modifying candidate for PD-related neurodegeneration.

## 1. Introduction

Parkinson’s disease (PD) is a chronic progressive neurodegenerative disorder clinically characterized by bradykinesia, resting tremor, rigidity, and postural instability. Neuropathologically, PD is defined by progressive degeneration of dopaminergic neurons in the substantia nigra pars compacta (SNpc), marked depletion of striatal dopamine, and dysfunction of basal ganglia motor circuitry. Despite advances in symptomatic management, no currently available therapy effectively halts the underlying neurodegenerative process. Oxidative stress, mitochondrial dysfunction, neuroinflammation, and neurotransmitter imbalance are widely recognized as major contributors to PD progression [1,2,3].

Rotenone (RT), a hydrophobic pesticide and potent inhibitor of mitochondrial complex I, is widely used to establish experimental PD models because it reproduces multiple behavioral, biochemical, and neurochemical abnormalities resembling those observed in PD [4,5,6,7]. RT-induced neurotoxicity is associated with enhanced oxidative and nitrosative stress, impaired mitochondrial bioenergetics, inflammatory activation, apoptotic signaling, and disruption of nigrostriatal neurotransmission [4,5,6,7]. Nevertheless, the unilateral intranigral RT model used here should be interpreted primarily as a toxin-induced nigrostriatal injury model with Parkinsonism-like features rather than a complete model of progressive idiopathic PD. Accordingly, the RT model provides a useful platform for evaluating compounds with antioxidant, anti-inflammatory, mitochondria-protective, and neurorestorative properties, while translational interpretation should remain appropriately cautious [4,6,7].

Loganin (LG), an iridoid glycoside derived mainly from *Cornus officinalis*, has been reported to possess antioxidant, anti-inflammatory, anti-apoptotic, and neuroprotective activities in a variety of experimental systems [8,9]. Notably, LG has shown protective effects in models of dopaminergic injury, including improvement of neurochemical deficits, attenuation of glial activation, and modulation of apoptosis- and autophagy-related signaling [8]. LG was selected for the present study because RT-induced nigrostriatal injury involves several convergent pathological cascades, including redox imbalance, mitochondrial dysfunction, neuroinflammation, apoptosis, and monoaminergic disruption. Compared with agents targeting only one downstream event, LG may be pharmacologically relevant because its reported activity profile overlaps with multiple components of RT-induced neuronal injury [8,9]. Given the multifactorial nature of PD, an agent capable of modulating redox imbalance, mitochondrial dysfunction, inflammation, apoptosis, and neurochemical disturbances may offer meaningful neuroprotection [8,9].

A further mechanistic consideration of the present study is antioxidant defense signaling. Because oxidative stress is a central component of RT-induced neurotoxicity, modulation of endogenous cytoprotective responses may contribute importantly to neuroprotection. Nrf2/antioxidant response element-related signaling is one candidate pathway because it regulates several antioxidant and detoxifying responses. In parallel, striatal catecholamine deficiency is a core neurochemical feature of PD and provides functional evidence of nigrostriatal injury [2,5,7]. Therefore, assessment of oxidative stress, antioxidant status, and striatal catecholamine homeostasis is important for defining the neuroprotective profile of candidate compounds [5,7].

To strengthen the pharmacological relevance of the study, selegiline (SL) was included as a reference antiparkinsonian treatment [10], whereas trigonelline (TG) was used as a pharmacological probe of antioxidant signaling [11,12]. TG has been reported to interfere with Nrf2 activation and antioxidant response-related gene expression in experimental systems. Therefore, TG co-treatment was used to explore whether the effects of LG were sensitive to disruption of antioxidant signaling. Because this approach is pharmacological and indirect, TG-related findings were interpreted as supportive rather than definitive pathway evidence. The present study was therefore designed to determine whether LG attenuates Parkinsonism-like abnormalities induced by a single unilateral stereotaxic RT lesion in rats and whether such effects are associated with modulation of oxidative stress, mitochondrial dysfunction, neuroinflammation, apoptosis, and nigrostriatal neurochemical disturbances.

## 2. Materials and Methods

### 2.1. Animals

Male Wistar rats (270–320 g; approximately 3 months old) were used in the present study. Animals were housed in groups of three in Plexiglas cages under standard laboratory conditions (22 ± 3 °C; 12 h light/dark cycle, lights on at 07:00) with free access to food and water. All experimental procedures were conducted in accordance with the Guide for the Care and Use of Laboratory Animals issued by the U.S. National Institutes of Health and were approved by the Institutional Animal Care and Use Committee of the National Taiwan University College of Medicine (IACUC approval no. 20220825). Behavioral assessments were performed during the animals’ active phase, between 19:00 and 23:00.

### 2.2. Chemicals and Reagents

Loganin (LG; purity ≥ 97%; CAS No. 18524-94-2; product code 36483), rotenone (RT; purity ≥ 98%; CAS No. 83-79-4; product code 557368-1GMCN), trigonelline (TG; CAS No. 535-83-1; product code 1686411-20MG), selegiline (SL; CAS No. 14611-52-0; product code 1611900-200MG), dimethyl sulfoxide (DMSO; CAS No. 67-68-5; product code D2650-100ML), polyethylene glycol (PEG; CAS No. 25322-68-3; product code 202398-250G), and phosphate-buffered saline (PBS; product code 806552-1L) were purchased from Sigma-Aldrich (St. Louis, MO, USA). Reagents used for biochemical and mitochondrial assays, including those for thiobarbituric acid reactive substances (TBARS), reduced glutathione (GSH), superoxide dismutase (SOD), catalase (CAT), and apoptosis-related assays, were obtained from standard commercial sources. Commercial kits or reagents for tumor necrosis factor-α (TNF-α), interleukin-1β (IL-1β), and interleukin-6 (IL-6) determinations were also used. Standards and reagents for catecholamine and amino acid analyses were obtained from KRISHGEN BioSystem (Whittier, CA, USA).

RT was freshly prepared immediately before stereotaxic injection by dissolving rotenone in DMSO:PEG (1:1, *v*/*v*) to obtain the required concentration for intranigral administration. A total volume of 2 μL RT solution was injected into the right SNpc in each RT-lesioned rat. Sham-operated rats received an equal volume of the corresponding DMSO:PEG vehicle. LG and SL were freshly prepared in double-distilled water for oral gavage on each day of administration. The working concentrations of LG and SL were adjusted according to the individual body weight of each animal to deliver the intended doses of LG at 3, 10, or 30 mg/kg and SL at 10 mg/kg in a fixed gavage volume of 2 mL/kg. TG was freshly dissolved in normal saline for intraperitoneal injection and administered at 10 mg/kg in an injection volume adjusted according to body weight. All drug solutions were prepared freshly on the day of use and protected from prolonged light exposure whenever applicable.

### 2.3. Experimental Design and Drug Treatment

After 7 days of acclimatization, rats were randomly assigned to twelve groups (*n* = 8 per group; total number of animals = 96) using a simple computer-generated randomization sequence: control (C), sham (S), RT (R), sham + LG 3 mg/kg (S + L3), sham + LG 10 mg/kg (S + L10), sham + LG 30 mg/kg (S + L30), RT + LG 3 mg/kg (R + L3), RT + LG 10 mg/kg (R + L10), RT + LG 30 mg/kg (R + L30), RT + TG + LG 10 mg/kg (R + T + L10), RT + TG + LG 30 mg/kg (R + T + L30), and RT + SL 10 mg/kg (R + SL). The sample size of *n* = 8 per group was selected based on prior RT-induced Parkinsonism studies using behavioral and biochemical endpoints and was intended to balance statistical sensitivity with animal welfare considerations [5,13]. A formal a priori power calculation was not performed. Group allocation was documented before treatment initiation, and coded animal identifiers were used during behavioral testing and sample analysis to reduce allocation-related bias.

A unilateral nigral lesion was induced on Day 0. Rats in the RT-lesioned groups received a single stereotaxic injection of RT into the right SNpc, whereas sham-operated rats received the corresponding vehicle. Control animals did not undergo stereotaxic surgery. LG was administered by oral gavage once daily from Day 1 to Day 21 at doses of 3, 10, or 30 mg/kg in a fixed volume of 2 mL/kg. These doses were selected to provide a low-, middle-, and high-dose range for evaluating dose-related neuroprotective effects, based primarily on previous preclinical efficacy studies of LG and related neuroprotective models rather than on a dedicated toxicological or pharmacokinetic optimization study performed in the present work. The 21-day post-lesion treatment period was selected to allow sufficient time for RT-induced behavioral, oxidative, mitochondrial, inflammatory, apoptotic, and neurochemical alterations to develop and to evaluate the repeated-treatment effects of LG during the post-lesion phase. This duration is also consistent with subacute treatment windows commonly used in experimental Parkinsonism studies assessing behavioral and biochemical endpoints. Sham + LG groups were included to assess whether LG alone produced detectable adverse effects under non-lesioned conditions. TG was administered intraperitoneally at 10 mg/kg, 30 min before LG, from Day 1 to Day 21. SL was administered by oral gavage at 10 mg/kg once daily from Day 1 to Day 21 as a reference antiparkinsonian treatment with monoamine oxidase-B inhibitory activity.

**Figure 1:**
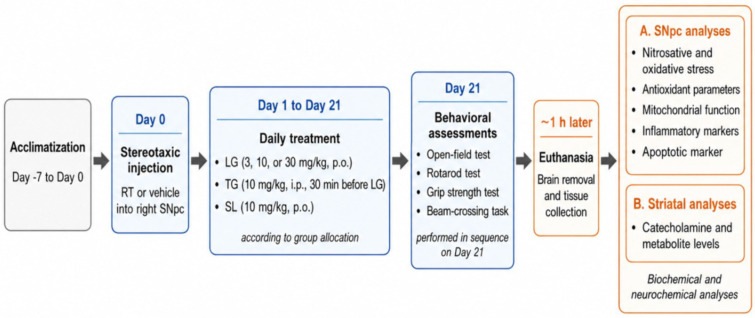
Diagrammatic representation of the experimental schedule.

Behavioral tests were performed on Day 21 in a fixed order from less stressful to more demanding motor tasks: open-field test, rotarod test, grip strength test, and beam-crossing task. The open-field test required approximately 12 min per animal, including a 2-min acclimation period followed by 10 min of recorded locomotor activity. The rotarod test required approximately 5–10 min per animal, depending on the latency to fall and repeated trials. Grip strength measurement required approximately 3–5 min per animal, and the beam-crossing task required approximately 5–10 min per animal, including habituation and test trials. Animals were allowed to rest between tests whenever needed to reduce fatigue and carryover effects. The total behavioral assessment period for each animal was approximately 30–40 min. To minimize observer bias, each animal was assigned an arbitrary code, and behavioral assessments were conducted by two investigators blinded to treatment allocation. Approximately 1 h after completion of behavioral testing, animals underwent euthanasia and tissue collection as described in Section 2.10. The SNpc and striatum were microdissected on ice and stored at −80 °C until further biochemical, mitochondrial, inflammatory, apoptotic, and neurochemical analyses. For these assays, sample tubes were coded before analysis, and data acquisition was performed using coded samples whenever feasible (Figure 1).

After 7 days of acclimatization, rats underwent stereotaxic intranigral injection of RT or vehicle on Day 0. LG, TG, or SL treatments were administered from Day 1 to Day 21 according to group allocation. Behavioral assessments, including the open-field test, rotarod test, grip strength test, and beam-crossing task, were performed on Day 21. Approximately 1 h after behavioral testing, animals were euthanized, and the SNpc and striatum were collected for biochemical, mitochondrial, inflammatory, apoptotic, and neurochemical analyses. RT, rotenone; LG, loganin; TG, trigonelline; SL, selegiline; SNpc, substantia nigra pars compacta; p.o., oral gavage; i.p., intraperitoneal.

### 2.4. Stereotaxic Injection of Rotenone

On Day 0, rats assigned to stereotaxic surgery were anesthetized with ketamine (80 mg/kg, i.p.) and xylazine (10 mg/kg, i.p.) and placed in a stereotaxic apparatus. After confirmation of adequate anesthesia, a midline scalp incision was made, and a burr hole was drilled over the target site. RT was dissolved in DMSO:PEG (1:1, *v*/*v*), and a single unilateral injection of RT solution (2 μL) was delivered into the right SNpc at a rate of 0.2 μL/min, following established stereotaxic RT lesion procedures used in experimental Parkinsonism-like models [4,5,7]. The stereotaxic coordinates for the SNpc were anteroposterior (AP), 5.3 mm; mediolateral (ML), 2.0 mm; and dorsoventral (DV), 7.5 mm, relative to bregma [14]. After injection, the needle was kept in place for 5 min to minimize reflux and was then slowly withdrawn. Sham-operated rats underwent the same surgical procedure but received an equal volume of DMSO:PEG vehicle instead of RT. Control animals did not undergo stereotaxic surgery. This unilateral intranigral lesion paradigm was used to generate reproducible toxin-induced nigrostriatal injury and Parkinsonism-like motor deficits, not to fully reproduce the chronic progressive course of idiopathic PD.

### 2.5. Measurement of Body Weight

Body weight was recorded on Day 1 and Day 21. Body weight change (%) was calculated for each animal using the following formula: body weight change (%) = [(body weight on Day 21 − body weight on Day 1)/body weight on Day 1] × 100.

### 2.6. Open-Field Test

Spontaneous locomotor activity was evaluated using a wooden open-field apparatus measuring 100 × 100 × 40 cm, with the floor divided into 25 equal squares. Illumination was provided by a 40 W white bulb positioned 150 cm above the apparatus. Each rat was placed individually in the center of the arena and allowed to explore for 12 min. The first 2 min were considered a habituation period and were not included in the analysis. Locomotor activity was calculated as the total number of floor-square crossings recorded during the final 10 min. A crossing was counted when all four paws of the animal entered a new square. The apparatus was cleaned between trials to eliminate olfactory cues [5].

### 2.7. Rotarod Test

Motor coordination and balance were assessed using a rotarod apparatus. Animals were pre-trained before formal testing. During the test, each rat was placed on a rotating rod (diameter, 7 cm; speed, 25 rpm), and the latency to fall was recorded. A cut-off time of 180 s was applied for each trial. The mean value of repeated trials was used for statistical analysis [15].

### 2.8. Grip Strength Measurement

Forelimb grip strength was measured using a digital grip strength meter (Chatillon, Greensboro, NC, USA) [16]. Each rat was allowed to grasp the metal grid with its forepaws and was then gently pulled backward until it released the grid. The maximal force exerted before release was recorded in kgf. The mean value obtained from repeated measurements was used as the grip strength score for each animal.

### 2.9. Beam-Crossing Task

Motor coordination and balance were further evaluated using a beam-crossing task [15]. The apparatus consisted of two circular platforms (8 cm in diameter) connected by a wooden beam measuring 120 cm in length, 2.0 cm in width, and 0.5 cm in thickness. The beam was elevated 50 cm above the floor, and a sawdust-filled box was placed underneath to prevent injury in case of falling. Before testing, each rat was allowed to explore the apparatus for 5 min to habituate to the elevated beam. During the test, the animal was placed on one platform and allowed to traverse the beam to the opposite platform. The time required to cross the beam and the number of foot slips were recorded.

### 2.10. Tissue Collection and Preparation of Homogenates

Approximately 1 h after completion of the behavioral assessments on Day 21, animals were euthanized by CO_2_ inhalation using a gradual-fill method according to the approved IACUC protocol. Death was confirmed by cessation of respiration and absence of reflexes before tissue collection. The brains were rapidly removed, and the SNpc and striatum were carefully dissected on ice, weighed, and homogenized in 0.1 M phosphate buffer (pH 7.4). Homogenates were centrifuged at 10,000× *g* for 15 min, and the resulting supernatants were collected for biochemical assays. Separate tissue aliquots were reserved for mitochondrial isolation and neurotransmitter determinations.

### 2.11. Assessment of Nitrosative and Oxidative Stress

Nitrite accumulation in SNpc homogenates was measured as an index of nitric oxide production using the Griess reaction [17]. Equal volumes of tissue supernatant and Griess reagent containing 0.1% N-(1-naphthyl)ethylenediamine dihydrochloride, 1% sulfanilamide, and 2.5% phosphoric acid were mixed and incubated for 10 min at 25 °C in the dark. Absorbance was measured at 540 nm, and nitrite concentration was calculated from a standard curve.

Lipid peroxidation was determined using the TBARS assay as described by Ohkawa et al. [18]. Results were expressed as nmol malondialdehyde (MDA)/mg protein after normalization to a 1,1,3,3-tetraethoxypropane standard.

### 2.12. Measurement of Antioxidant Parameters

Reduced glutathione (GSH) levels were determined by the Ellman method [19]. Briefly, homogenates were precipitated with 10% trichloroacetic acid and reacted with Ellman’s reagent containing 5,5′-dithiobis(2-nitrobenzoic acid) in sodium citrate/phosphate buffer (pH 8.0). After centrifugation, absorbance was measured at 412 nm, and results were expressed as nmol GSH/mg tissue.

Superoxide dismutase (SOD) activity was measured based on inhibition of adrenaline auto-oxidation to adrenochrome at 480 nm [20]. One unit of SOD activity was defined as the amount of enzyme producing approximately 50% inhibition of adrenaline auto-oxidation, and results were expressed as U/mg tissue.

Catalase (CAT) activity was determined by monitoring decomposition of hydrogen peroxide at 240 nm every 10 s for 1 min [21]. One unit of CAT activity was defined as the amount of enzyme required to decompose 1 mmol of peroxide per min at 25 °C and pH 7.0, and results were expressed as U/mg tissue.

### 2.13. Assessment of Mitochondrial Function and Respiratory Complex Activities

Mitochondria were isolated from SNpc tissue by differential centrifugation, with slight modifications of a previously described method [22]. Briefly, tissue samples were homogenized in ice-cold isolation buffer containing 215 mM mannitol, 75 mM sucrose, 0.1% *w/v* bovine serum albumin, 20 mM HEPES, and 1 mM ethylene glycol-bis(β-aminoethyl ether)-N,N,N′,N′-tetraacetic acid (EGTA), adjusted to pH 7.2. The homogenate was centrifuged at 1300× *g* for 5 min at 4 °C to remove nuclei and debris. The supernatant was then centrifuged at 14,000× *g* for 10 min at 4 °C to obtain the mitochondrial pellet. The pellet was washed in EGTA-free isolation buffer and centrifuged again at 14,000× *g* for 10 min at 4 °C. Mitochondrial protein content was determined by the Lowry method using bovine serum albumin as the standard [23].

Mitochondrial function was assessed by the MTT reduction assay and expressed as μg formazan formed/min/mg protein [24]. Mitochondrial membrane potential (MMP) was evaluated using tetramethylrhodamine methyl ester (TMRM). Briefly, mitochondrial suspensions were incubated in assay buffer followed by addition of TMRM for 15 min at 37 °C. After incubation, samples were centrifuged and the pellet was resuspended in phosphate-buffered saline. Fluorescence intensity was measured at an excitation wavelength of 535 ± 10 nm and an emission wavelength of 580 ± 10 nm [25].

Complex I activity was estimated by catalytic oxidation of NADH and expressed as nmol NADH oxidized/min/mg protein [26]. Complex II activity was determined by tetrazolium reduction and expressed as μmol formazan produced/min/mg protein [27]. Complex IV activity was measured using reduced cytochrome c as substrate and expressed as nmol cytochrome c oxidized/min/mg protein [28]. Complex V activity was assessed by ATP hydrolysis and expressed as nmol ATP hydrolyzed/min/mg protein [29].

### 2.14. Measurement of Neuroinflammatory and Apoptotic Markers

TNF-α, IL-1β, and IL-6 levels in SNpc homogenates were quantified using commercial rat ELISA kits (KRISHGEN BioSystem, Whittier, CA, USA) according to the manufacturers’ instructions. Cytokine concentrations were calculated from standard curves and expressed as pg/mg protein.

Caspase-3 activity was measured using a colorimetric assay based on cleavage of a caspase-specific peptide substrate linked to p-nitroaniline (pNA) (R&D Systems, Minneapolis, MN, USA). Released pNA was quantified spectrophotometrically at 405 nm, and results were expressed as nmol pNA/mg protein.

### 2.15. Determination of Striatal Catecholamine Levels

Striatal levels of dopamine (DA), norepinephrine (NE), serotonin (5-HT), and selected monoamine metabolites, including the dopamine metabolites 3,4-dihydroxyphenylacetic acid (DOPAC) and homovanillic acid (HVA), and the serotonin metabolite 5-hydroxyindoleacetic acid (5-HIAA), were measured by high-performance liquid chromatography (HPLC) with electrochemical detection, as previously described [30]. The HPLC system consisted of a high-pressure isocratic pump, a 20 μL manual injector, a C18 reverse-phase column, and an electrochemical detector. The mobile phase comprised sodium citrate buffer (pH 4.5) and acetonitrile (87:13, *v*/*v*), containing 10 mM citric acid, 25 mM NaH_2_PO_4_, 25 mM EDTA, and 2 mM 1-heptane sulfonic acid. Electrochemical detection was performed at +0.75 V with a sensitivity range of 5–50 nA. Separation was achieved at a flow rate of 0.8 mL/min, and 20 μL of each sample was injected.

On the day of analysis, frozen striatal samples were thawed and homogenized in 0.2 M perchloric acid. Homogenates were centrifuged at 12,000× *g* for 10 min, and the resulting supernatants were filtered through 0.22 μm nylon filters before HPLC injection. Concentrations were calculated from standard curves generated using standard solutions in the range of 10–100 ng/mL. Results were expressed as pg/mg tissue for DA, NE, and 5-HT, and as ng/mg tissue for DOPAC, HVA, and 5-HIAA.

### 2.16. Statistical Analysis

The sample size of n = 8 per group was selected based on previous RT-induced Parkinsonism and neuroprotective efficacy studies using comparable behavioral and biochemical endpoints, while also considering animal welfare and experimental feasibility. A formal a priori power calculation was not performed. The individual animal was considered the experimental unit for all behavioral, biochemical, mitochondrial, inflammatory, apoptotic, and neurochemical analyses. All data are presented as the mean ± SEM. Statistical analyses were performed using GraphPad Prism version 8.3.0 (GraphPad Software, San Diego, CA, USA). Comparisons among groups were performed using one-way analysis of variance (ANOVA) followed by Tukey’s multiple-comparison test. A *p* value < 0.05 was considered statistically significant.

## 3. Results

### 3.1. LG Attenuated RT-Induced Body Weight Change

**Figure 2:**
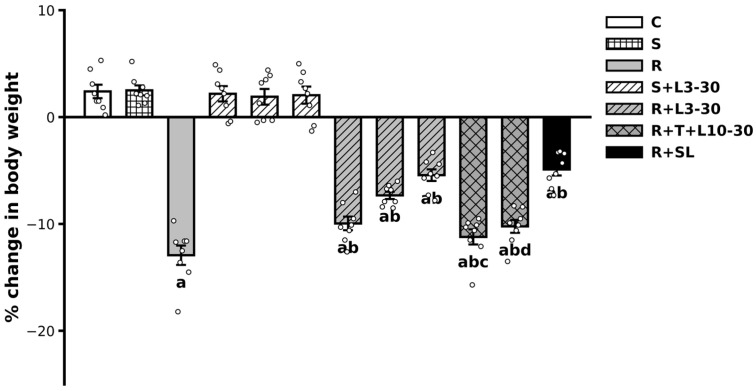
Effects of LG on RT-induced body weight change. Percentage body weight change was calculated between Day 1 and Day 21. Data are expressed as the mean ± SEM, with individual dots representing values from individual animals (*n* = 8/group). Statistical analysis was performed using one-way ANOVA followed by Tukey’s post hoc test. “a” *p* < 0.001 vs. C; “b” *p* < 0.001 vs. R; “c” *p* < 0.001 vs. R+L10; “d” *p* < 0.001 vs. R+L30.

Body weight change (Figure 2) differed significantly among groups (F(11,84) = 87.70, *p* < 0.001). The control and sham groups showed comparable body weight changes (2.40 ± 0.63% and 2.50 ± 0.46%, respectively), and no significant differences were observed in the sham + LG groups (S+L3: 2.18 ± 0.72%, S+L10: 1.90 ± 0.74%, and S+L30: 2.05 ± 0.80%), indicating that LG alone did not affect body weight under non-lesioned conditions. In contrast, RT lesioning markedly reduced body weight relative to the control and sham groups (R: −12.93 ± 0.91% vs. C: 2.40 ± 0.63% and S: 2.50 ± 0.46%; both *p* < 0.001). LG treatment attenuated RT-induced body weight loss, with more evident protection at 10 and 30 mg/kg (R+L3: −9.95 ± 0.64%, R+L10: −7.33 ± 0.34%, and R+L30: −5.44 ± 0.54%). The effect reached significance at 10 mg/kg and 30 mg/kg versus the RT group (both *p* < 0.001), whereas the 3 mg/kg dose did not reach significance (*p* = 0.0733). TG co-administration attenuated this protective effect, as reflected by greater body weight loss in R+T+L10 (−11.21 ± 0.71%) and R+T+L30 (−10.22 ± 0.60%) than in the corresponding LG-treated groups (*p* = 0.0035 and *p* = 0.0001, respectively). SL also significantly improved body weight change compared with the RT group (R+SL: −4.90 ± 0.56%, *p* < 0.001).

### 3.2. LG Improved Motor and Behavioral Performance in RT-Lesioned Rats

**Figure 3:**
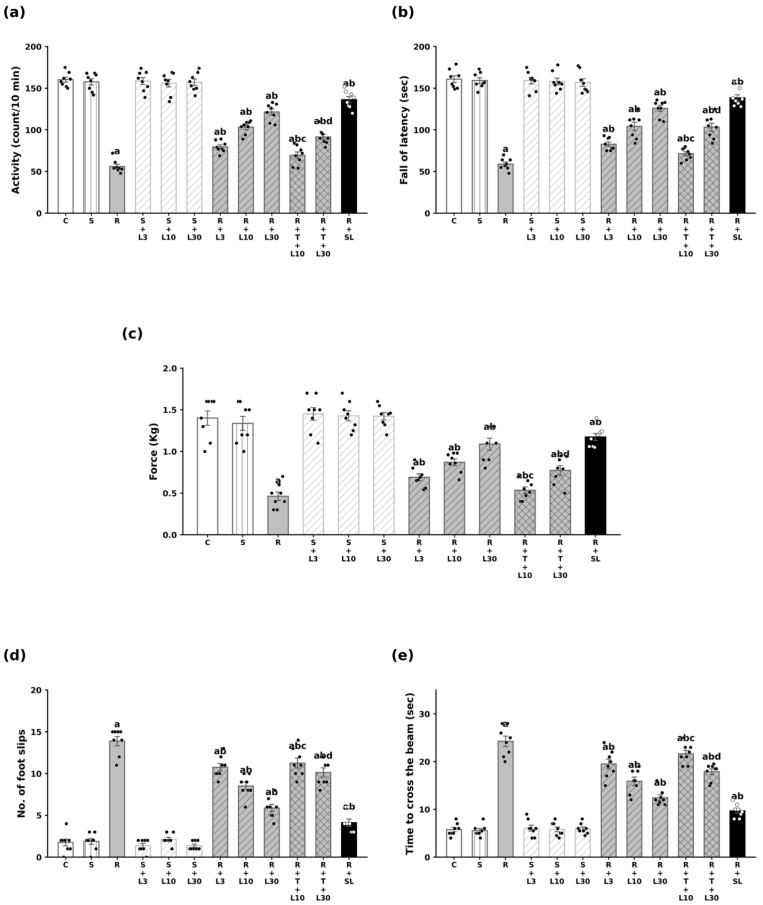
Effects of LG on RT-induced motor and behavioral impairments. (**a**) Open-field activity. (**b**) Rotarod performance. (**c**) Forelimb grip strength. (**d**) Number of foot slips in the beam-crossing task. (**e**) Time required to cross the beam. Data are expressed as the mean ± SEM, with individual dots representing values from individual animals (*n* = 8/group). Statistical analysis was performed using one-way ANOVA followed by Tukey’s post hoc test. “a” *p* < 0.001 vs. C; “b” *p* < 0.001 vs. R; “c” *p* < 0.001 vs. R+L10; “d” *p* < 0.001 vs. R+L30.

Motor and behavioral performance differed significantly among groups in the open-field (Figure 3a), rotarod (Figure 3b), grip strength (Figure 3c), and beam-crossing tests (Figure 3d,e) (open field: F(11,84) = 120.98, *p* < 0.001; rotarod: F(11,84) = 99.13, *p* < 0.001; grip strength: F(11,84) = 37.65, *p* < 0.001; foot slips: F(11,84) = 114.63, *p* < 0.001; beam-crossing time: F(11,84) = 105.36, *p* < 0.001). No significant differences were detected among the control, sham, and sham + LG groups in any of these measures, indicating that LG alone did not alter basal motor performance.

RT lesioning produced marked motor impairment, as evidenced by reduced spontaneous locomotor activity in the open-field test (R: 56.12 ± 2.60), shortened latency to fall in the rotarod test (R: 58.88 ± 2.45 s), decreased forelimb grip strength (R: 0.46 ± 0.05 kgf), increased foot slips in the beam-crossing task (R: 13.88 ± 0.55), and prolonged beam-crossing time (R: 24.25 ± 1.08 s), compared with the control and sham groups (all *p* < 0.001). LG treatment significantly ameliorated these deficits, with stronger effects generally observed at 10 and 30 mg/kg. In the open-field test, locomotor activity increased to 79.62 ± 2.38, 103.25 ± 2.75, and 121.50 ± 3.62 in the R+L3, R+L10, and R+L30 groups, respectively. In the rotarod test, latency to fall increased to 82.88 ± 2.64 s, 104.25 ± 4.96 s, and 125.88 ± 3.47 s, respectively. Grip strength was similarly improved to 0.69 ± 0.04 kgf, 0.87 ± 0.04 kgf, and 1.09 ± 0.07 kgf, while beam-crossing performance improved as indicated by reduced foot slips (10.75 ± 0.45, 8.50 ± 0.46, and 5.88 ± 0.44) and shorter crossing times (19.50 ± 1.02 s, 15.88 ± 0.87 s, and 12.44 ± 0.59 s) in the corresponding groups.

TG co-administration attenuated the beneficial effects of LG across these behavioral endpoints. Compared with the corresponding LG-treated groups, R+T+L10 and R+T+L30 animals showed lower open-field activity (69.50 ± 4.00 and 91.50 ± 3.32), shorter rotarod latency (71.38 ± 2.54 s and 103.14 ± 5.58 s), weaker grip strength (0.54 ± 0.04 kgf and 0.77 ± 0.06 kgf), more foot slips (11.25 ± 0.59 and 10.12 ± 0.55), and longer beam-crossing times (21.62 ± 0.73 s and 17.88 ± 0.60 s). SL also significantly improved lesion-induced motor deficits, as reflected by increased open-field activity (136.25 ± 3.76), prolonged rotarod latency (138.38 ± 3.71 s), improved grip strength (1.17 ± 0.04 kgf), reduced foot slips (4.12 ± 0.44), and shortened beam-crossing time (9.69 ± 0.49 s) relative to the RT group (all significant vs. R).

### 3.3. LG Reduced Nitrosative Stress and Lipid Peroxidation in the SNpc

**Figure 4:**
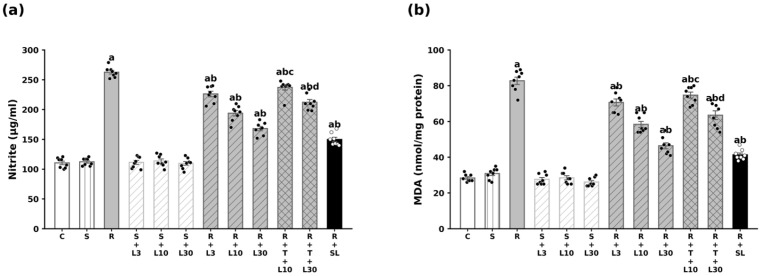
Effects of LG on RT-induced nitrosative and oxidative stress in the SNpc. (**a**) Nitric oxide (NO) levels. (**b**) Thiobarbituric acid reactive substances (TBARS) levels. Data are expressed as the mean ± SEM, with individual dots representing values from individual animals (*n* = 8/group). Statistical analysis was performed using one-way ANOVA followed by Tukey’s post hoc test. “a” *p* < 0.001 vs. C; “b” *p* < 0.001 vs. R; “c” *p* < 0.001 vs. R+L10; “d” *p* < 0.001 vs. R+L30.

Nitric oxide (Figure 4a) and TBARS levels (Figure 4b) differed significantly among groups (NO: F(11,84) = 238.15, *p* < 0.001; TBARS: F(11,84) = 154.14, *p* < 0.001). RT lesioning markedly increased both NO and TBARS levels relative to the control and sham groups, indicating enhanced nitrosative and oxidative damage in the SNpc. LG treatment significantly attenuated these elevations in RT-lesioned rats, with more evident protection at 10 and 30 mg/kg. Co-administration of TG weakened the effect of LG on both parameters, whereas SL also significantly reduced RT-induced increases in NO and TBARS. No significant differences were observed among the control, sham, and sham + LG groups, indicating that LG alone did not alter these indices under non-lesioned conditions.

### 3.4. LG Restored Endogenous Antioxidant Defenses in the SNpc

**Figure 5:**
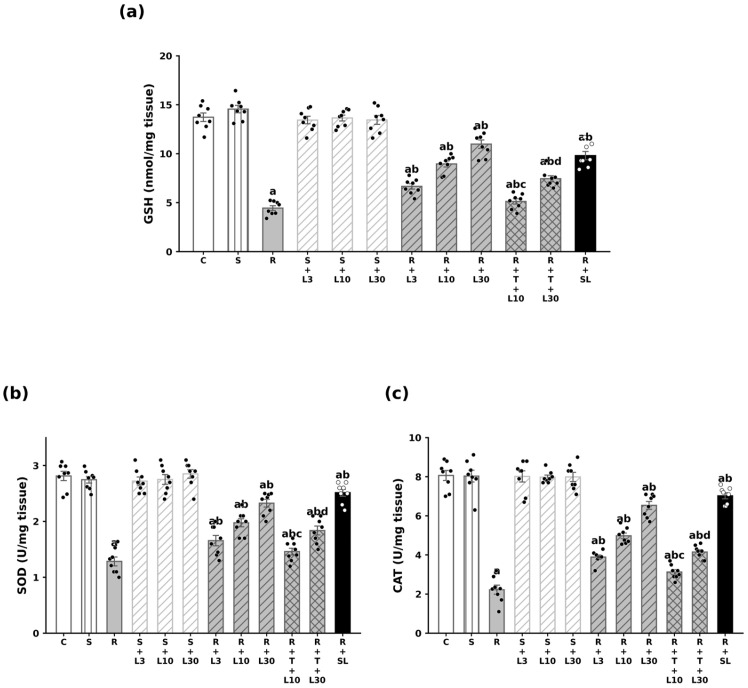
Effects of LG on endogenous antioxidant defenses in the SNpc. (**a**) Reduced glutathione (GSH) levels. (**b**) Superoxide dismutase (SOD) activity. (**c**) Catalase (CAT) activity. Data are expressed as the mean ± SEM, with individual dots representing values from individual animals (*n* = 8/group). Statistical analysis was performed using one-way ANOVA followed by Tukey’s post hoc test. “a” *p* < 0.001 vs. C; “b” *p* < 0.001 vs. R; “c” *p* < 0.001 vs. R+L10; “d” *p* < 0.001 vs. R+L30.

Significant group differences were observed in reduced glutathione (GSH) (Figure 5a), superoxide dismutase (SOD) (Figure 5b), and catalase (CAT) (Figure 5c) levels in the SNpc (GSH, F(11,84) = 103.15, *p* < 0.001; SOD, F(11,84) = 57.89, *p* < 0.001; CAT, F(11,84) = 120.53, *p* < 0.001). No significant differences were detected among the control, sham, and sham + LG groups, indicating that LG alone did not materially affect basal antioxidant status under non-lesioned conditions. In contrast, RT lesioning markedly reduced all three antioxidant parameters relative to the control and sham groups.

LG treatment significantly restored endogenous antioxidant defenses in RT-lesioned rats in a dose-dependent pattern, with more evident effects at 10 and 30 mg/kg. GSH levels increased from 4.45 ± 0.24 in the R group to 6.66 ± 0.28, 8.95 ± 0.31, and 10.97 ± 0.43 in the R+L3, R+L10, and R+L30 groups, respectively. Similarly, SOD levels increased from 1.28 ± 0.08 in the R group to 1.66 ± 0.09, 1.98 ± 0.07, and 2.33 ± 0.07, whereas CAT levels increased from 2.22 ± 0.23 to 3.89 ± 0.11, 4.98 ± 0.14, and 6.53 ± 0.20 across the corresponding LG-treated groups. Co-administration of TG significantly attenuated the restorative effects of LG, as shown by lower GSH, SOD, and CAT levels in the R+T+L10 and R+T+L30 groups than in the corresponding LG-treated groups. SL also significantly improved all three antioxidant parameters compared with the R group.

### 3.5. LG Preserved Mitochondrial Function in the SNpc

**Figure 6:**
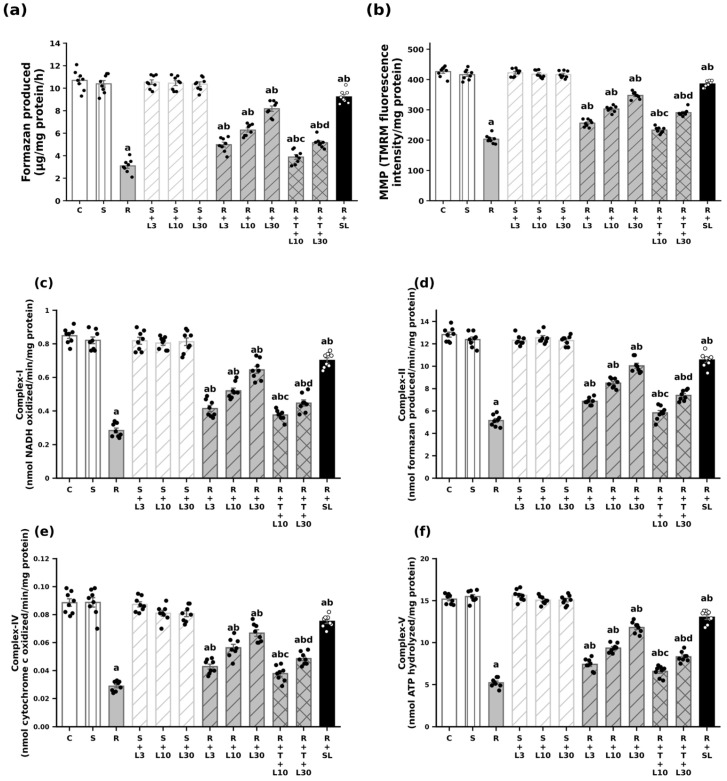
Effects of LG on mitochondrial dysfunction in the SNpc. (**a**) MTT reduction. (**b**) Mitochondrial membrane potential (MMP), assessed using tetramethylrhodamine methyl ester (TMRM) fluorescence. (**c**) Complex I activity. (**d**) Complex II activity. (**e**) Complex IV activity. (**f**) Complex V activity. Data are expressed as the mean ± SEM, with individual dots representing values from individual animals (*n* = 8/group). Statistical analysis was performed using one-way ANOVA followed by Tukey’s post hoc test. “a” *p* < 0.001 vs. C; “b” *p* < 0.001 vs. R; “c” *p* < 0.001 vs. R+L10; “d” *p* < 0.001 vs. R+L30.

Mitochondrial indices differed significantly among groups, including MTT reduction (Figure 6a), mitochondrial membrane potential (MMP) (Figure 6b), and the activities of complexes I (Figure 6c), II (Figure 6d), IV (Figure 6e), and V (Figure 6f) (MTT, F(11,84) = 166.18, *p* < 0.001; MMP, F(11,84) = 335.79, *p* < 0.001; complex I, F(11,84) = 136.77, *p* < 0.001; complex II, F(11,84) = 233.24, *p* < 0.001; complex IV, F(11,84) = 101.83, *p* < 0.001; complex V, F(11,84) = 316.28, *p* < 0.001). No significant differences were observed among the control, sham, and sham + LG groups. In contrast, RT lesioning markedly impaired mitochondrial function in the SNpc, as reflected by reduced MTT reduction, MMP, and respiratory complex activities relative to the control and sham groups.

LG treatment significantly ameliorated these mitochondrial deficits in RT-lesioned rats, with more evident protection at 10 and 30 mg/kg. MTT values increased from 3.10 ± 0.21 in the R group to 4.99 ± 0.22, 6.29 ± 0.18, and 8.18 ± 0.24 in the R+L3, R+L10, and R+L30 groups, respectively. Similarly, MMP increased from 203.38 ± 4.97 to 256.25 ± 4.12, 302.88 ± 3.49, and 347.75 ± 3.99, whereas complex I activity increased from 0.28 ± 0.01 to 0.42 ± 0.02, 0.52 ± 0.02, and 0.65 ± 0.02 across the corresponding LG-treated groups. Parallel improvements were also observed for complex II, complex IV, and complex V activities. TG co-administration significantly attenuated these protective effects, whereas SL significantly improved all mitochondrial parameters compared with the R group.

### 3.6. LG Suppressed Neuroinflammatory and Apoptotic Responses in RT-Lesioned Rats

**Figure 7:**
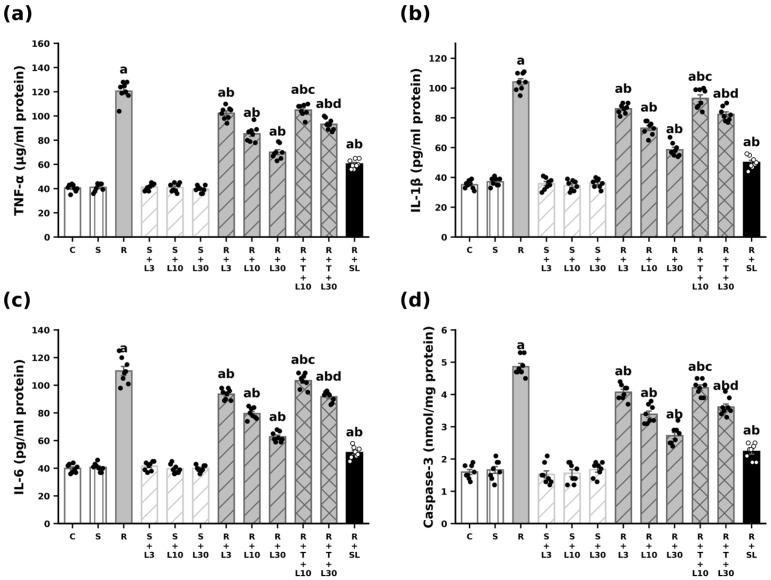
Effects of LG on inflammatory and apoptotic markers in the SNpc. (**a**) Tumor necrosis factor-α (TNF-α). (**b**) Interleukin-1β (IL-1β). (**c**) Interleukin-6 (IL-6). (**d**) Caspase-3. Data are expressed as the mean ± SEM, with individual dots representing values from individual animals (*n* = 8/group). Statistical analysis was performed using one-way ANOVA followed by Tukey’s post hoc test. “a” *p* < 0.001 vs. C; “b” *p* < 0.001 vs. R; “c” *p* < 0.001 vs. R+L10; “d” *p* < 0.001 vs. R+L30.

Significant group differences were observed in TNF-α (Figure 7a), IL-1β (Figure 7b), IL-6 (Figure 7c), and caspase-3 (Figure 7d) levels in the SNpc (TNF-α, F(11,84) = 325.03, *p* < 0.001; IL-1β, F(11,84) = 298.47, *p* < 0.001; IL-6, F(11,84) = 320.05, *p* < 0.001; caspase-3, F(11,84) = 167.46, *p* < 0.001). No significant differences were observed among the control, sham, and sham + LG groups. In contrast, RT lesioning markedly increased inflammatory cytokine and caspase-3 levels relative to the control and sham groups, indicating robust inflammatory and apoptotic activation in the SNpc.

LG treatment significantly attenuated these RT-induced changes in a dose-dependent manner, with more evident effects at 10 and 30 mg/kg. TNF-α levels decreased from 120.62 ± 2.78 in the R group to 102.38 ± 1.82, 85.38 ± 2.24, and 70.12 ± 2.02 in the R+L3, R+L10, and R+L30 groups, respectively. Likewise, IL-1β levels decreased from 104.12 ± 2.08 to 86.12 ± 1.16, 73.12 ± 1.62, and 58.62 ± 1.59; IL-6 levels from 110.38 ± 3.27 to 93.62 ± 1.35, 79.62 ± 1.43, and 62.75 ± 1.24; and caspase-3 levels from 4.86 ± 0.10 to 4.08 ± 0.08, 3.39 ± 0.10, and 2.73 ± 0.10 across the corresponding LG-treated groups. TG co-administration significantly attenuated the protective effect of LG, whereas selegiline significantly reduced inflammatory cytokine and caspase-3 levels compared with the R group.

### 3.7. LG Attenuated Striatal Catecholamine Disturbances

**Figure 8:**
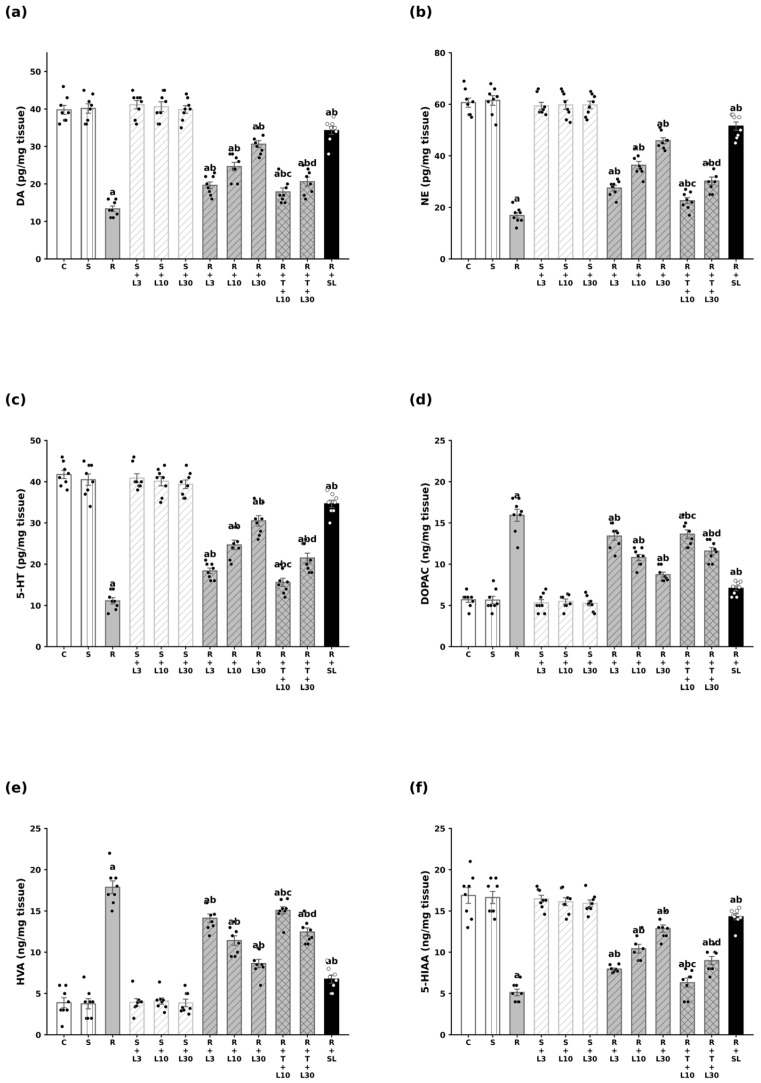
Effects of LG on striatal catecholamine homeostasis. (**a**) Dopamine (DA). (**b**) Norepinephrine (NE). (**c**) Serotonin (5-HT). (**d**) 3,4-dihydroxyphenylacetic acid (DOPAC). (**e**) Homovanillic acid (HVA). (**f**) 5-hydroxyindoleacetic acid (5-HIAA). Data are expressed as the mean ± SEM, with individual dots representing values from individual animals (*n* = 8/group). Statistical analysis was performed using one-way ANOVA followed by Tukey’s post hoc test. “a” *p* < 0.001 vs. C; “b” *p* < 0.001 vs. R; “c” *p* < 0.001 vs. R+L10; “d” *p* < 0.001 vs. R+L30.

Significant group differences were observed in striatal DA (Figure 8a), NE (Figure 8b), 5-HT (Figure 8c), DOPAC (Figure 8d), HVA (Figure 8e), and 5-HIAA (Figure 8f) levels (DA, F(11,84) = 91.02, *p* < 0.001; NE, F(11,84) = 131.11, *p* < 0.001; 5-HT, F(11,84) = 111.07, *p* < 0.001; DOPAC, F(11,84) = 82.05, *p* < 0.001; HVA, F(11,84) = 94.12, *p* < 0.001; 5-HIAA, F(11,84) = 68.29, *p* < 0.001). No significant differences were detected among the control, sham, and sham + LG groups. In contrast, RT lesioning markedly decreased striatal DA, NE, 5-HT, and 5-HIAA levels, while increasing DOPAC and HVA levels, indicating pronounced neurochemical disturbance in the nigrostriatal system.

LG treatment significantly ameliorated these alterations in RT-lesioned rats, with more evident effects at 10 and 30 mg/kg. DA levels increased from 13.38 ± 0.73 in the R group to 19.62 ± 0.91, 24.62 ± 1.15, and 30.62 ± 0.94 in the R+L3, R+L10, and R+L30 groups, respectively. Similar dose-related improvements were observed for NE, which increased from 16.88 ± 1.08 to 27.50 ± 1.05, 36.38 ± 1.45, and 45.88 ± 1.12, and for 5-HT, which increased from 11.12 ± 0.77 to 18.38 ± 0.68, 24.68 ± 1.15, and 30.50 ± 1.27 across the corresponding LG-treated groups. In parallel, 5-HIAA increased from 5.12 ± 0.40 in the R group to 7.95 ± 0.14, 10.43 ± 0.53, and 12.86 ± 0.44 following LG treatment. By contrast, the lesion-induced elevations in DOPAC and HVA were significantly reduced by LG. DOPAC decreased from 15.93 ± 0.72 in the R group to 13.41 ± 0.51, 10.81 ± 0.38, and 8.74 ± 0.30, whereas HVA decreased from 17.88 ± 0.77 to 14.12 ± 0.50, 11.41 ± 0.58, and 8.64 ± 0.51 in the R+L3, R+L10, and R+L30 groups, respectively. TG co-administration significantly attenuated these beneficial effects, whereas SL significantly improved the overall neurochemical profile compared with the R group.

## 4. Discussion

The present study demonstrated that LG attenuated Parkinsonism-like abnormalities induced by a single unilateral stereotaxic RT lesion in rats. LG improved motor performance, reduced oxidative and nitrosative stress, restored endogenous antioxidant defenses, preserved mitochondrial function, suppressed inflammatory and apoptotic responses in the SNpc, and ameliorated striatal catecholamine disturbances. Together, these findings support a multi-target neuroprotective effect of LG in RT-induced nigrostriatal injury.

The unilateral intranigral RT model produced behavioral, biochemical, mitochondrial, inflammatory, apoptotic, and neurochemical abnormalities consistent with nigrostriatal dysfunction. RT is a mitochondrial complex I inhibitor and has been widely used to model PD-relevant oxidative stress, mitochondrial impairment, and nigrostriatal neurochemical alterations [4,5,6,7]. However, this model should be interpreted primarily as a toxin-induced Parkinsonism-like injury model rather than a complete model of progressive human PD [4,7]. LG improved spontaneous locomotion, motor coordination, forelimb grip strength, and beam-crossing performance, suggesting partial preservation of motor system function rather than a purely nonspecific symptomatic effect. The inclusion of sham + LG groups further supported the tolerability of the selected dose range, as LG alone did not produce detectable adverse effects on body weight or basal motor performance under the present experimental conditions. Nevertheless, this observation should be regarded as preliminary safety-related evidence rather than formal toxicological validation.

The protective effects of LG were associated with attenuation of several interconnected injury mechanisms. Oxidative stress, mitochondrial dysfunction, neuroinflammation, and apoptosis are major contributors to PD-related neurodegeneration and are also central features of RT-induced nigrostriatal injury [1,2,3,4,5,6,7]. In the present study, RT-induced oxidative stress, antioxidant depletion, mitochondrial dysfunction, inflammatory activation, and caspase-3-associated apoptotic signaling were mitigated by LG treatment. These findings are consistent with previous reports showing that LG possesses antioxidant, anti-inflammatory, anti-apoptotic, and neuroprotective properties in experimental neurological disease models [8,9]. Because oxidative injury, mitochondrial impairment, inflammation, and apoptosis can mutually reinforce nigrostriatal degeneration, the coordinated improvement of these endpoints supports a multi-target protective profile for LG rather than action through a single isolated pathway [1,3,7].

LG also improved striatal monoamine homeostasis, including dopamine and related metabolite disturbances, indicating that its biochemical effects were associated with preservation of neurochemical function in the nigrostriatal pathway. Striatal dopamine depletion and monoaminergic imbalance are key neurochemical features of PD and experimental nigrostriatal injury [1,2,7]. TG co-administration weakened several beneficial effects of LG. Since TG has been reported to interfere with antioxidant defense-related signaling, including Nrf2-associated responses [11,12], these findings suggest that antioxidant defense mechanisms may contribute to LG-mediated protection. However, this interpretation remains pharmacological and indirect because Nrf2 nuclear translocation, Keap1-Nrf2 pathway activation, and downstream targets such as HO-1, NQO1, and GCLC were not directly measured.

The protective profile of LG is consistent with other candidate neuroprotective agents evaluated in RT-related Parkinsonism models, including L-theanine, apigenin, and berberine, which have been associated with antioxidant, anti-inflammatory, mitochondrial-protective, or anti-apoptotic effects [5,6,13]. However, the present study extends this literature by evaluating LG across a broader endpoint panel within the same unilateral intranigral RT lesion paradigm, including behavioral performance, redox balance, mitochondrial complex activity, inflammatory cytokines, caspase-3 activity, and striatal catecholamine/metabolite profiles. This integrated design is important because RT-induced nigrostriatal injury is not driven by a single isolated downstream event; rather, oxidative stress, complex I dysfunction, inflammatory activation, apoptosis, and monoaminergic disturbance interact within the same injury cascade. Accordingly, the present data position LG as an iridoid glycoside with multi-target activity across behavioral, mitochondrial, inflammatory, apoptotic, and neurochemical endpoints. Direct head-to-head comparisons are still needed to determine whether LG offers superior efficacy, distinct pathway selectivity, or improved translational potential compared with other neuroprotective candidates.

SL served as a useful pharmacological reference in the present study. Although levodopa/carbidopa is the gold-standard symptomatic treatment for PD, selegiline was selected because it is a monoamine oxidase-B inhibitor relevant to monoamine metabolism, oxidative stress-related injury, mitochondrial dysfunction, and neurochemical endpoints [10,13]. Since this study focused on neuroprotection-related biochemical and neurochemical changes rather than acute dopaminergic replacement, SL was considered a mechanistically appropriate comparator. Future studies directly comparing LG with levodopa/carbidopa would help clarify its symptomatic versus neuroprotective potential.

Overall, the present findings indicate that LG exerts multi-target neuroprotective effects against RT-induced Parkinsonism-like abnormalities. These effects were associated with reduced oxidative injury, preserved mitochondrial function, suppressed inflammation and apoptosis, and improved striatal catecholamine homeostasis. Because pathway-specific validation and long-term translational assessments were not performed, these findings should be interpreted as evidence of association rather than definitive mechanistic proof.

## 5. Limitations

Several limitations should be acknowledged. First, the unilateral stereotaxic RT lesion model reproduces several PD-relevant pathological features but does not fully capture the progressive and multifactorial nature of human PD. Second, dopaminergic neuronal preservation was not histologically confirmed by tyrosine hydroxylase immunostaining or neuronal counting. Third, antioxidant defense-related signaling was inferred from TG co-administration and biochemical antioxidant endpoints, but direct pathway validation, including Nrf2 nuclear translocation and downstream targets such as HO-1, NQO1, and GCLC, was not performed. Fourth, the apoptotic analysis was limited to caspase-3 activity and did not include additional mitochondrial apoptotic markers. Fifth, only male rats were used, and long-term functional recovery, sustained neuroprotection, and disease-modifying potential were not assessed. Finally, dose selection was based primarily on prior preclinical efficacy ranges rather than dedicated toxicological, pharmacokinetic, or brain-exposure data. Future studies should incorporate histological validation, direct pathway assays, serum and tissue biomarker analyses, sex-inclusive designs, long-term endpoints, and pharmacokinetic/toxicological profiling to better define the translational significance of LG-mediated protection.

## 6. Conclusions

LG attenuated Parkinsonism-like abnormalities induced by a single stereotaxic RT lesion in rats. Its protective effects were associated with improved motor performance, reduced oxidative and nitrosative stress, preservation of mitochondrial function, suppression of inflammatory and apoptotic responses, and amelioration of striatal catecholamine disturbances. TG co-administration weakened these benefits, suggesting that antioxidant defense-related mechanisms may contribute to LG-mediated protection; however, direct pathway validation remains necessary. SL improved lesion-induced abnormalities and served as a useful pharmacological reference. Overall, these findings support further investigation of LG as a candidate neuroprotective agent for PD-related neurodegeneration, particularly in studies incorporating histological confirmation, direct pathway assays, serum biomarker analysis, pharmacokinetic/toxicological evaluation, sex-inclusive validation, and long-term functional endpoints.

## Data Availability

The data presented in this study are available from the corresponding author upon reasonable request.

## Abbreviations

The following abbreviations are used in this manuscript:

CAT	Catalase
DA	Dopamine
DOPAC	3,4-dihydroxyphenylacetic acid
GSH	Reduced glutathione
HVA	Homovanillic acid
IL-1β	Interleukin-1β
IL-6	Interleukin-6
LG	Loganin
NE	Norepinephrine
PD	Parkinson’s disease
RT	Rotenone
SL	Selegiline
SNpc	Substantia nigra pars compacta
SOD	Superoxide dismutase
Str	Striatum
TBARS	Thiobarbituric acid reactive substances
TG	Trigonelline
TNF-α	Tumor necrosis factor-α
5-HIAA	5-hydroxyindoleacetic acid
5-HT	Serotonin
ANOVA	Analysis of variance
HPLC	High-performance liquid chromatography
MDA	Malondialdehyde
MMP	Mitochondrial membrane potential
MTT	3-(4,5-dimethylthiazol-2-yl)-2,5-diphenyltetrazolium bromide
Nrf2	Nuclear factor erythroid 2-related factor 2
SEM	Standard error of the mean
